# Legislation for Midwives

**Published:** 1900-05-26

**Authors:** 


### LEGISLATION FOR MIDWIVES.

By a Correspondent.

### IV.—ITS EFFECT ON THE MIDWIFE.

I now pass to the consideration of the manner in which this Bill is likely to affect the midwives themselves.

The intention of its promoters is to raise the whole calling by conferring upon those who exercise it special privileges, and admitting none to fellowship without a special qualification. Everything, however, depends on the standard of qualification which is aimed at, and on this point the Bill throws no light. It is, indeed, hardly possible that it should lay down any hard and fast rule. During two years of grace the standard must necessarily be lowered to admit all who are now in practice, and who can show themselves to be not egregiously ignorant. With this provision no one would quarrel. The old rough and ready midwife will die out in the course of a few years, and, as I have before said, the majority of them are even now fairly efficient. When the work of licensing begins, however, it is to be expected that many cases will arise in poor districts where the qualifications of the practising midwife are too inadequate to pass muster, and I look in vain in the Bill for any provision for giving free instruction in such a contingency. I am speaking now of localities sufficiently populated to keep a midwife employed, but too poor to attract the services of anyone not settled in the neighbourhood. Unless one of the local people be trained for the work, the licence would have to be conferred on one of those "incapables" whom it is desired to abolish, and the cost of training a midwife is considerable. A three months' course of midwifery in a lying-in hospital costs from about £26 in London to £12 or £15 in Edinburgh, Dublin, and certain provincial towns. Extern instruction can be had for less, but it is doubtful if anything short of actual training in residence would eradicate old errors and impart the new system to uneducated persons. Money will certainly be required for this work, and where legislation has been invoked, it will not do to rely on charitable effort.

The most important point, however, in this legislation from the midwives' point of view is the qualification expected from those in what may be called normal practice. There is nothing in this Bill to indicate that its promoters are aware of any higher standard than is implied by the possession of "a certificate of midwifery from the Royal College of Physicians in Ireland, or from the Obstetrical Society of London." The certificate of the latter body, familiarly known as the "L.O.S.," is the aim of nearly all women who qualify themselves to practise as midwives, and the London Obstetrical Society has done much by the holding of its examinations to encourage women to undergo a regular course of training. But it must be remembered that the last 20 years, which have brought about a complete revolution in obstetric surgery, have rendered it increasingly clear that dirt is the enemy chiefly to be dreaded, while "Dame Nature," as an old midwife quaintly observes, "is chiefest of midwives." The life of mother and infant depend, in fact, in ordinary cases, mainly, if not entirely, on the exactness with which antiseptic precautions are observed by the nurse. In cases where complications ensue, the two lives may probably depend on the nurse's accuracy of observation, leading her promptly to summon medical aid, and on her judgment and presence of mind until such aid arrive. None of these qualities, however, in the nurse can be tested by examination or imparted by oral instruction. The nurse may know by rote all the various ways in which she must guard against septic infection, but nothing but daily discipline and the continual performance of her duties under the eye of a trained nurse will make scrupulous cleanliness a matter of habit with her. I have shown that the lying-in hospitals have long been providing education for midwives on these lines. But the term of training is admittedly too short. In three months, which is the minimum under which a certificate can be granted, a woman may be so far trained as to be enabled to obtain the L.O.S. certificate. She will have seen, and herself assisted in, sufficient cases to enable her to perform the ordinary duties of a midwife, and she will be strongly impressed with the best methods of guarding against dirt. It would, however, be disastrous to the midwives' calling if this were set as the highest standard attainable.

There is at present working in various parts of the country a large body of midwives, who have, in addition to this special training in a lying-in hospital, undergone two, three, and even four years training in general hospitals, and these women have undoubtedly some claim to higher consideration than has been afforded hitherto by the provisions of the Bill for their regulation. The term "trained nurse" in its highest sense implies not merely the possession of a considerable amount of technical knowledge and of skill in many difficult manipulations, but subjection to a discipline so all-pervading that observation, promptitude, and obedience to exactest precaution have become a second nature. It is now universally acknowledged that nothing short of a three years' course of instruction in well-equipped hospitals is sufficient for the education of a well-trained nurse, and it will hardly be denied that a very broad line of demarcation lies between the qualifications of such a trained nurse midwife, and those of the ordinary holder of a certificate of three months' instruction. It would be a strange, and not very creditable, anomaly, if the highest standard for a midwife were settled by law at a qualification far below what any surgeon would deem desirable in the nurse who was to carry out his orders in the simplest surgical case.

I have already shown that it is not possible to make a very high standard compulsory for all midwives. But it is no less of the utmost importance that the better class of women should be encouraged to remain in the calling by the establishment of some superior qualification at which any might aim who desired to improve their status. The value of some such "counsel of perfection" was early perceived in France, where two classes of midwives have always been recognised; the first class holding their licence for life from a special "faculty," and enjoying the liberty of practising where they would. It will probably be found that this class represents the really well-trained element in French midwifery. The second class hold provincial licences, renewable every year, and are restricted from practice in any but their special locality. They are, in fact, exactly on the footing to which it is sought to reduce the whole body of highly-trained midwives in this country, and there is only too much reason to fear that the effect must be equally deplorable.

In the interests of English midwifery we believe it to be essential that any Bill for regulating the calling should recognise a higher class of trained nurse midwives, qualified by at least three years' training, in addition to their midwifery course, whose licence should be granted for life, subject, of course, to withdrawal in case of misconduct, and who should have full liberty to follow their calling in any part of the kingdom.

